# Orientation towards the vernacular and style-shifting as language behaviours in speech of first-generation Polish migrant communities speaking Norwegian in Norway

**DOI:** 10.3389/fpsyg.2024.1330494

**Published:** 2024-08-14

**Authors:** Kamil Malarski, Chloe Castle, Witosław Awedyk, Magdalena Wrembel, Isabel Nadine Jensen

**Affiliations:** ^1^Department of Sociolinguistics and Discourse Studies, Faculty of English, Adam Mickiewicz University in Poznań (AMU), Poznań, Poland; ^2^Department of Language and Culture, Arctic University in Tromsø (UiT), Tromsø, Norway; ^3^Institute of Linguistics, Faculty of Humanities, University of Szczecin (US), Szczecin, Poland; ^4^Department of Contemporary English Language, Faculty of English, Adam Mickiewicz University in Poznań (AMU), Poznań, Poland

**Keywords:** L3 dialect acquisition, L3 Norwegian, style-shifting, vernacular, multilingualism

## Abstract

This study describes the patterns of dialect use among L3 Norwegian speakers born in Poland who have migrated to Norway. We collected the data in the form of sociolinguistic interviews recorded in Tromsø and Oslo, two different dialect regions, in order to examine potential differences in acquisition of two dissimilar dialects in Norwegian by L3 speakers. The analyses focus on dialectal and accentual variation in their speech, and whether frequency of dialect use is dependent on selected sociocultural factors. We have found that some speakers, especially those scoring high for overall dialect use, also display style-shifting, i.e. they use dialect features from the region more frequently in unscripted speech as opposed to in more formal speech styles elicited through reading tasks or the wordlist reading tasks. This demonstrates that language learners are capable of developing sensitivity towards the vernacular form in an L3. Moreover, it shows that first-generation migrant communities in fact may be capable of developing their L2/L3/L4 language competencies in a similar way to L1 speakers, including at the level of sociolinguistic variation.

## 1 Introduction

Dialect acquisition and dialectal variation in a foreign language has not often been discussed in investigations of multilingual acquisition. In many descriptions, the topic has been neglected because a learner (a non-L1 user of a language) is expected to speak using standard forms which they have been taught from the language learning material (Husby, 2019; Balasubramanian, 2022; Milojičić, 2023). The choice and the use of the dialect is, however, vitally important for the social meanings which language communicates, i.e. it matters whether one uses the standard or the vernacular forms. A lack of attention to the topic may also be due to the popular misunderstanding that since second- or third-language structures are processed differently on a neural, cognitive and socio-cultural level, one will never attain the pragmatic competence which would enable them to fully participate in the social and cultural contexts which are constructed through the language learnt later in their lives.

In order to address the identified research gap, this article describes the acquisition and sociolinguistics of dialect use among Polish speakers of Norwegian, who were born in Poland but reside permanently in Norway. Specifically, we investigate which dialectal features are acquired first, and which last, in Norwegian spoken as an L2 and L3 by our informants, as well as to what extent these patterns reflect style-shifting (in terms of speech style or modality).

Polish people are the largest migrant group in Norway. As of 2023, there were 107,442 Polish immigrants living in Norway, and 16,583 Norwegian-born people with Polish parents (Statistics Norway, 2023a, b). Polish people relocate to Norway often for economic purposes. They mostly settle in cities, hence we have selected two urban dialects to describe how they may be acquired by this particular L3 community. Their perspective in dialect acquisition in Norwegian may be especially relevant in the light of the differences of the dialect landscapes of Poland and Norway (see Garbacz, 2014). In the former, dialects have considerably lower status in society and standard forms are encouraged. It is worth testing, therefore, whether this L3 community may be willing to use dialectal forms during the interviews.

In this article, we first explain the dialectal variation present in Norway. We then briefly explore the concept of a language ‘standard’ and its role in acquisition research. This is followed by a section on dialect acquisition in the L1 and Ln, and speaker orientation towards the vernacular. We then discuss dialectal variation and style shifting, before moving to the sociolinguistic situation for migrant communities in Norway. Subsequently, we outline our research questions and methodology, including information on participants, equipment, recordings, and procedure and analysis. Thereafter, we discuss results in terms of general dialect use, linguistic and sociocultural predictors, style-shifting, a dialect feature hierarchy, and individual variability. We describe these in light of our research questions, followed by limitations and conclusions.

## 2 Dialectal variation in Norway

Norwegian is a language characterised by high dialectal variation, the origins of which date back to the Old Norse period when, at the turn of the second millennium, national and regional variations among the North Germanic tribes began to emerge (Torp, 1998: 34ff). In the broadest sense, we could differentiate between four larger dialectal areas which are Western Norwegian – Vestnorsk, Eastern Norwegian – Østnorsk, The Trøndelag dialect – Trøndersk, and Northern Norwegian – Nordnorsk (Kristoffersen, 2000). The traditional division of Norwegian dialects may be perceived as somewhat arbitrary, as it does not account for many linguistic phenomena underlying the presence of an individual dialect feature in a given area (*cf.* Helleland and Papazian, 2005; Mæhlum and Røyneland, 2023: 27ff). Consequently, alternative classifications, such as that of Sandøy (1985) where he identifies as many as 12 dialect groups, have been under debate among Scandinavian dialectologists (*cf.* Mæhlum and Røyneland, 2023: 27ff). In other words, there is much more variation within each region. In addition to the spoken regional varieties, there are two *written* standards of Norwegian, Bokmål and Nynorsk. Bokmål is the primary language of the majority of Norwegian school children, whereas Nynorsk is the primary language of 11.6% of Norwegian school children (Statistics Norway, 2022). In both cases, children are taught the other non-primary standard as a second language form from grade eight.

It is difficult to identify a *spoken* ‘standard’ for Norwegian. It has sometimes been associated with the variety used in Oslo (capital; e.g. Kristoffersen, 2000), yet Johnsen (2015) shows in their metaanalysis that this may not be entirely accurate. In addition, the use of dialects is an important part of Norwegian culture. Røyneland (2017) describes how dialects work as an index of one’s background and identity. They describe how changing one’s dialect or mixing dialects (*knote*) is perceived as negative in the eyes of many Norwegians, as the dialects should rather remain “pure” and unchanged (Røyneland, 2017: 95–96). Speaking a non-Oslo dialect is in many contexts viewed as more Norwegian. This is mirrored in findings reported by the author where Norwegians tend to assess boys with foreign appearance as less *Norwegian* when they use the Oslo dialect compared to when they use other dialects (Røyneland, 2017: 101). The closest variety associated with the standard is the Oslo dialect, or, broadly, the South-East Norwegian dialect (Johnsen, 2015). This similarity between the Oslo dialect and what may be conceived of as a spoken standard ‘standardtalemål’ (Mæhlum, 2009; Sandøy, 2009) in Norwegian is an important consideration in the current study, wherein we assess acquisition of the Oslo dialect and the Tromsø dialect by L3 Norwegian speakers.

Following the traditional approach to the mapping of the Norwegian dialects, regional varieties can be identified by the set of the so-called primary and secondary distinctive features. The opposition between high and low tone, together with *tjukk* ‘l’ (retroflex flap [ɽ]), retroflexion and *jamvekt* (‘even stress’; a prosodic feature that originates from Old Norse and affects the stress patterns of two-syllable words, particularly verbs), constitute the set of primary distinctive features between the four groups of Norwegian dialects: Eastern Norwegian, Western Norwegian, Trøndelag Norwegian, and Northern Norwegian (Kristoffersen, 2000; Mæhlum and Røyneland, 2023). Following this taxonomy, East Norwegian and Trøndelag Norwegian are classified as low-tone dialects while West Norwegian and North Norwegian are defined as high-tone dialects (Kinn and Kulbrandstad, 2023). As the above set does not suffice to account for the dialect variation of Norwegian, one may also resort to some other phonological dialect features such as palatalization, mono- or diphthongization or initial word stress in words of foreign origin. Furthermore, individual dialects can also be identified by a selection of morphophonemic features, among which the most salient are: personal pronouns (in particular first- person singular and plural, third-person feminine singular, as well as second- and third-person plural), the negative form *ikke* (variable with *ikkje*), the definite ending of feminine nouns, vowel change in the present tense forms of strong verbs and, dative endings in the noun paradigm (*cf.* Mæhlum and Røyneland, 2023). While some of these morphophonemic features do not concern the two dialects selected for the present study (the dative), other (personal pronouns and *ikke*/*ikkje* in particular) will affect the findings of the study conducted among the Polish L3 speakers in Oslo and Tromsø.

Some other selected dialect features in the two regions are presented in Table 1 (Tromsø) and Table 2 (Oslo). The dialect features described in Tables 1, 2 are also subject to variation within the regions due to both dialect levelling and more dialect contact, as described in Røyneland (2020). For instance, research on Northern Norwegian spoken varieties finds a development towards less palatalisation (Bull, 1996; Sætermo, 2011), and less lowering of vowels (Hårstad, 2010). Another relevant point is that retroflexion, as described in Table 2, is dominant in Norway, with the exception of the western regions.

**Table 1:** Selected Tromsø dialect features.

Tromsø
palatalisation on /t/, /d/, /l/, /n/ in words like *vann*, *fjell*
lowering of /i/ to /e/, e.g. in *fisk*
lowering of /e/ to /æ/, e.g. in *sett*
Pronouns *dokker* / *dokkers* for *dere* / *deres* (‘you’ (pl) / ‘yours’ (pl))
Interrogatives *ka* / *kor* / *kæm*

**Table 2:** Selected Oslo dialect features.

Oslo
retroflexion for /rt./, /rl/, /rd/, /rn/, /rs/ - > [ʈ], [ɭ], [ɖ], [ɖ], [ɳ], [ʂ]
*tjukk* /l/ (retroflex flap [ɽ]), e.g. in *sola*
tone 1 – tone 2 phonemic distinction: ⟨ɑˋ⟩ for accent 1, ⟨ɑˆ⟩ for accent 2
-a ending in praeteritum and present perfect participles in the so-called -a verb class
*-a* ending in the definite forms of the (potentially) feminine nouns

## 3 Dialect acquisition and orientation towards the vernacular

The capacity of a person to acquire a dialect, irrespective of whether in their first or second language, is an ability related to acquiring a language as a whole (Chevrot and Ghimenton, 2018; Oschwald et al., 2018). There are only a few accounts which juxtapose a language and a dialect in this context (see Oschwald et al., 2018), stating that a dialect is a form of the ‘standard’ language differing in grammar and/or pronunciation features. Such a concept may be misleading, since every user of a language (L1 or not) speaks in a variety of a language, and ‘standard’ varieties are essentially the standardised forms of regional dialects as they have evolved, e.g. in England or in Norway (Trudgill, 2011; Johnsen, 2015). Notably, in both countries the dialects understood as the most standard come from the South-East, i.e. the regions surrounding the capital cities.

The question of the spoken ‘standard’ and the ‘dialect’ in Norway is rather complex and multi-layered. On the one hand, it seems that lay users of the language often emphasise that all dialects are equal, and hence few social situations require accommodation of one’s dialect. On the other, the same ideologised language attitudes seem to be suppressive towards those L1 Norwegian speakers who would wish to adapt their dialect to the new local forms after having migrated within the country (Sætermo and Sollid, 2021). This may be seen in connection to the so-called emic and epic perspectives in perceptual dialectology (Cramer, 2018). What will be considered as the dialect (or the standard) in everyday interactions (emic perspective) may be a little dissimilar with what has been theorised as such by the researchers (etic perspective).

Also important to mention in the context of Norwegian is bidialectalism, i.e. speaking in two dialects of the same language (Tagliamonte and Molfenter, 2007; Nycz, 2015; Wu et al., 2016; Nycz, 2019). The discussions around bidialectism often make a distinction in whether code switching occurs between two regional dialects, or between one’s regional dialect and the standardised variety (Trumper, 1989), and while, e.g. L1 speakers of Italian would code-switch depending on the social situation (Trumper, 1989), this is much less frequent in Norway (Nesse, 2023). In Norway, the dominating variety when it comes to the media, theatre, TV etc. has traditionally been Standardtalemål (also often understood as Urban Eastern Norwegian but this term is sometimes used in a wider context)^1^, meaning that Norwegian speakers may still widely meet and acquire this variety irrespective of their own dialect which is then indicative of bidialectalism (Lundquist and Vangsnes, 2018); although it must be noted that the media has become much more inclusive in recent years towards the use of the dialect (see also Røyneland and Lanza, 2023). An important contribution to the contextualisation and social meanings conveyed through bidialectalism among L1 Norwegian speakers is van Ommeren (2016). It reports on how switching between the dialect forms and the more standard forms are a conscious socio-psychological process whereby Norwegian speakers build their social personas against language ideologies pertaining in a given community of speakers. A similar more recent study describes code switching between the Northern Norwegian and the South-Eastern Urban Norwegian by Tromsø children (Strand, 2022) showing how they style shift from the local forms into the South-Eastern forms, e.g. when playing. The use of dialect is vitally important in the discussion of the language use among the members of migrant communities in Norway because their dialects are intertwined with how they are viewed within the society overall. For instance, people representing foreign to Norway ethnicities are viewed more positively when speaking with an Oslo dialect than when speaking other dialects, e.g. Bergen or Valdres, as migrant groups are often expected to speak with an accented Norwegian or Standard Eastern Norwegian; they, however, are still not treated equally with ethnically native Norwegians who use the Oslo dialect in terms of the perceived dynamism (Røyneland and Jensen, 2020).

The research questions addressed in this article are based on the assumption that Ln speakers may in fact develop sensitivity towards the vernacular, and that the process is connected with the acquisition of pragmatics at the level of language processing and production and as the last component of the language structure (after semantics, syntax, pronunciation etc.). One study investigating this among transnational immigrants and their children finds that the first generation *does not* seem to adapt to phonological categories spoken in the region where they live, but the next generation does acquire this variation, and thus rejects their parent’s idiosyncratic accentual patterns (Labov, 2014). This vernacular reorganisation is instigated by a new source of social contact, namely, entering school and transitioning from primarily adult interaction to interaction with older peers (Denis et al., 2019). Labov (2001), citing Johnson and Newport (1989) suggests that this vernacular reorganisation stabilises at age 17, corresponding to the age at which the ability to acquire L1 syntactic intuitions has effectively ceased. However, for adults learning a new language, the development of sociolinguistic variation in the L2/Ln is understudied. Indeed, if living in the country where the L2 is spoken, they now have a new source(s) of social contact, which could possibly trigger vernacular reorganisation in the L2. Outside of the English-language context, it is also unknown as to whether they are learning the standard before moving towards the vernacular, or starting with both.

To the best of our knowledge, there is a small pool of studies indicating that Ln speakers *can and do* use dialect forms, and do so variably depending on sociolinguistic factors. In their study of second dialect acquisition in a second language, Gnevsheva et al. (2022) found that L2 speakers of English were more likely than L1 speakers to select Australian (as opposed to American) lexical items to label pictures after having lived in Australia. The L2 speakers of English were L1 Russian speakers who had started learning British English in their home country. Notably, this tendency to choose more Australian lexical items holds even for L2 American English D2 (second dialect) Australian English participants when compared with L1 American English D2 Australian English speakers. This suggests that L2 speakers are actually more likely than L1 speakers to be sensitive to and use different dialectal features, perhaps due to the fact that one’s L1D1 serves more often as an identity marker (*cf.* Siegel, 2021 on L1D1 acquisition of Australian English). Another study investigating L2D2 acquisition is that of Drummond (2013), who shows that migrants with a Polish background in Manchester can and do acquire and use the Northern STRUT variant in their L2 English production, though they do so variably. Speakers are more likely to use this variant when they have a strong emotional relationship with a local person, or when they have particularly positive attitudes towards the region. We test comparable parameters in our Polish-born speakers from Oslo and Tromsø to see whether similar patterns may occur.

## 4 Style shifting

Use of the dialect is vitally important for conveying social meaning. In other words, it matters whether and when one uses standard forms and dialect forms and to what extent one switches between them, i.e. to what extent they style-shift. People typically use dialect forms in everyday interactions, i.e. while talking to family and closer friends, much more than in formal situations. The rates of divergence between the standard vs. dialect may be different for different languages and countries in Europe, which is dependent on many political, social (e.g. class structure), cultural and historical factors, e.g. in the Slavic languages landscape the standard is much more widely spoken than in Norway (Auer, 2011).

Popularisation of the sociolinguistic interview by Labov in his New York study (Labov, 1966) brought to light the extent to which variation occurs in a language across different language registers, or styles. Since then, this method has been adopted throughout sociolinguistics to capture the production of different linguistic variables and assess how differently they may be distributed in speech in different parts of the interview. What Labov suggested was that the more careful or formal the speech style is, the dialectal (or vernacular) features one produces. This has a lot to do with speech standardisation, school education and other social mechanisms allowing speakers of different languages to adapt the way they speak to different people and different social contexts, purely for the reasons of being understood more clearly and communicating more appropriately (Gooskens, 2018). The contexts closest to informal casual speech in a recorded interview are traditionally questions eliciting spontaneous answers, e.g. about one’s lives, memories and childhood (Milroy and Gordon, 2003: 66; Labov, 2006: 70–71; Tagliamonte and Molfenter, 2007: 37–40).

What we are looking for in speech production among L3 speakers has already been shown for L1 speakers of Norwegian. In their extensive analysis, Lundquist et al. (2020) presented evidence showcasing the situations wherein Tromsø high school students would resort to using the standard, and when the regional forms would instead be used on morphological, syntactic, phonological and lexical levels. In a controlled environment, they recorded the students’ speech in a few modes. The findings clearly show that Tromsø L1 speakers of Norwegian do style-shift, producing many more dialect features in unscripted spoken tasks compared to when reading texts, for example. Their strategies for style shifting differed, however, with reference to different parts of the grammar; namely, especially the production of syntactic structures was not subject to style-shifting as much as in other categories. Reversely, many dialectal morphological forms (e.g. *ka*, *kem*, *kor* as opposed to standard *hva*, *hvem*, *hvor*) were almost always preferred in the open speech tasks Lundquist et al. (2020).

## 5 Methodology

### 5.1 Aims and research questions

In the light of large dialect variation in Norway, as well as interesting social constraints under which the Polish migrant communities acquire the Norwegian dialects, we had phrased the following research questions before recruiting our informants:

1. How do L3 speakers acquire dialect features from the areas where they live?
2. How do they develop a sensitivity towards the dialect and do they use it differently in different speech registers or modalities (e.g. read vs. spoken)? If so, which dialect features are acquired earlier/later?
3. What are sociolinguistic predictors of dialect use? Does Norwegian proficiency or length of stay play a role?

We aimed to answer these questions by recruiting speakers in Oslo and Tromsø, two selected different dialect regions and assessing acquired dialect features with reference to variables such as the length of stay in Norway and proficiency in Norwegian.

### 5.2 Participants

Our informants comprised a group of 18 Polish-English-Norwegian speakers recorded in Oslo, and 18 recorded in Tromsø. The Oslo group included 16 female participants (all gender identities self-reported), and 2 male participants. The Tromsø group, on the other hand, included 13 female and 5 male participants. They all spoke Norwegian to an upper-intermediate or advanced level [as measured with the Norwegian Proficiency Test adapted from Language Trainers (2015)].

The Oslo group was quite uniform and displayed certain social characteristics. They had stayed in Norway for 10.8 years on average. They were aged between 31 and 63 (mean age = 40.05). Their average proficiency level for English was 18 / 24, and 25.2 / 28 for Norwegian. Many of them were engaged in higher-profile jobs; they ran their own companies (2), they were academics (3), teachers (7), engineers (2) etc. Many of them had strong ties with Polish culture and traditions. This is evidenced by the fact that a group of 13 people were recruited through a Polish Saturday school.

The Tromsø group seemed a little less uniform along social and economic scales. They were aged between 22 and 59. Their average length of stay in the Tromsø region was 4.9 years, and 6.5 years in Norway. The group was characterised with a very similar proficiency in Norwegian (average 25/28, as tested). The Tromsø group as a whole comprised academics (1), university students (9), artists (1), as well physical workers (7) from a more traditionally understood migrant community.

The participants were recruited through our extended circles and social media advertisements. They were remunerated for their participation. In the recruitment process, all were welcome regardless of their gender, ethnicity, religion or other social criteria. Their profiles are presented in Tables 3–6.

**Table 3:** Social and language characteristics for the Oslo group.

	Mean	Range
Age (years)	39.7	31–53
Residence in Norway (in years)	12.6	1–32
AoO NO	24	3–35
AoO EN	10	3–35
AoO PL	0	–
NO proficiency	25.6 (91%)	20–28 (B2 – C2)
EN proficiency	17.75 (71%)	7–24 (A2 – C1)
% Norwegian friends	36	0–80

**Table 4:** Profession characteristics for the Oslo group.

	Number
Administrative	2
Teacher	2
Cleaning services	1
Air traffic control and administration	1
Medicine and medicine related	3
Warehouse worker	1
Editorial work (e.g. in a publishing house)	1
HR	1
Managerial	1
Researcher (at university)	2
Engineering (industrial, environmental)	1
Interpreting	1

**Table 5:** Social and language characteristics for the Tromsø group—L1 Polish group.

	Mean	Range
Age (years)	33	22–59
Residence in Norway	6 years 6 months	1 year 9 months - 16 years
AoO NO	22–35 yrs	22–36+ yrs.
AoO EN	11–14 yrs	3–36+ yrs
AoO PL	0–2 yrs	0–2 yrs
NO proficiency	24.7 (88%)	12–28 (A2 - C1)
EN proficiency	19.2 (77%)	6–25 (A2 - C2)
% Norwegian friends	38.5	2–95

**Table 6:** Profession characteristics for the Tromsø group.

	Number
Student	9
Tradesperson	1
Cleaner	2
Hospitality	1
Retail	1
Artist	1
Managerial	2
Researcher (at university)	1

### 5.3 Data collection and analysis

This section outlines data collection, the applied procedures, as well as approaches to the subsequent analyses (Table 7). The data collection part lasted for about 45 min per person and included the sociolinguistic interview (c. between 15 and 35 min), followed by a sociodemographic questionnaire and proficiency tests in English and Norwegian. The sociolinguistic interview comprised three parts: a short interview in Polish, a short interview in English, and the main Norwegian component (see Table 8). The Norwegian part involved a text reading task of the Norwegian version of The North Wind and the Sun text (*Nordavinden og sola; spelled in Bokmål, progressive version*), followed by three semi-spontaneous tasks eliciting unscripted speech, i.e. narratives about free time activities, what they ate for breakfast, and daily routines. This was followed by a minimal pairs task involving production of word pairs which differ only in tone (e.g. *gjenta* / *jenta*), however this task was administered only to the Oslo group. Notably, most L1D1 (first dialect) Oslo speakers have the distinction, while in Tromsø many have minimal to no tonal distinction (Kristoffersen, 2000; Helleland and Papazian, 2005). Another difficulty is that the tone 2 feature in Norwegian is particularly difficult to learn for Ln speakers (e.g. Wetterlin, 2006, Haukland, 2016). The next task was a wordlist, which involved elicitation of pronunciation features such as retroflexion (Oslo), palatalization (Tromsø), vowel lowering (Tromsø), pronunciation of /r/ before /k/ (Tromsø). The material for the interview differed between Oslo and Tromsø with respect to the first wordlist, as different phonological categories were tested for these separate regions. The complete testing material which was administered to the participants can be seen in materials supplemented in the online repository.

**Table 7:** Sociolinguistic interview tasks in Oslo.

Order	Part
1	Reading passage (*Nordavinden og sola*)
2	Picture description task
3	Unscripted speech (*Fritid i Norge, ‘Free time in Norway’*)
4	Unscripted speech (*Beskriv på norsk din daglige* *Rutine*, ‘Tell us about your daily routine’)
5	Unscripted speech (*Hva spiser du til frokost?*, ‘What do you eat for breakfast?’)
6	Minimal pairs for tone distinction
7	Wordlist

**Table 8:** Sociolinguistic interview tasks in Tromsø.

Order	Part
1	Reading passage (*Nordavinden og sola*)
2	Picture description task
3	Unscripted speech (*Fritid i Norge, ‘Free time in Norway’*)
4	Unscripted speech (*Beskriv på norsk din daglige Rutine*, ‘Tell us about your daily routine’)
5	Unscripted speech (*Hva spiser du til frokost?*, ‘What do you eat for breakfast?’)
6	Wordlist

The administration of the tasks was as follows; they were displayed on a 14 or 15-inch monitor in the form of a ppt presentation. The word tokens in the wordlist were shown one at a time; whereas in the minimal pairs task, two at a time. The participants were allowed to operate the presentation on their own, while the interviewer was sitting next to them, sometimes getting involved in a conversation in the open conversation tasks if the interviewee directed them, in whichever language the participant chose. This was usually the language the interviewer introduced themselves in - there were three interviewers, namely, an L1 Polish speaker, an L1 English speaker, and an L1 Norwegian speaker. Interviewers tried to use the language of the task component as much as possible. Once the sociolinguistic interview was finished, the participants filled in three online questionnaires which included (1) a sociodemographic questionnaire, (2) a proficiency test for English (the Cambridge Proficiency Test), and (3) a proficiency test for Norwegian [adapted from Language Trainers (2015)]. The sociodemographic questionnaire and the Norwegian Proficiency test can be found in Appendices 1, 2, respectively.

The equipment used throughout the interviews was a Marantz™ PMD 661 portable recorder and a SHURE™ SM35 overhead unidirectional microphone attached to it with an XLR cable. Speech was recorded at 44.1 kHz, 16-bit depth rate and saved to mono sounds in the wave format. The recorders were plugged into electric sockets while recording. The interviews were all recorded in quiet spaces in Oslo and Tromsø; including classrooms, a conference room and office rooms in university buildings.

The data analysis comprised the following steps. All the tasks were designed for the identification of dialectal features from the two selected regions, Oslo and Tromsø, respectively. In order to verify whether and to what extent the informants had acquired and used the dialect in the interview, each interview was listened to and rated by two Norwegian linguists familiar with both dialects. In each part of the interview, the given speaker was rated on a scale from 0 to 6, i.e. 0 when they used no dialect features from the regions, and 6 if they were using them in all applicable contexts. Later, for each speaker, their general dialect score (0–6) was calculated as the average of scores assigned to individual tasks. Hence, the dialect score was calculated based on dialect features encompassing lexical, phonological and morphosyntactic structures and how they were produced in the context of a sociolinguistic interview.

## 6 Results

### 6.1 General dialect use

The general dialect scores were calculated on the basis of the component scores marked in each task of the interview. Table 9 presents the descriptive results for dialect use across regions and styles. The data coming from each task represents the decreasing formality of speech style, i.e. from the wordlist where the most formal speech style was elicited to the unscripted speech task where the participants answered freely to everyday questions. What is noticeable is that on average the Oslo speakers displayed higher scores for dialect (M = 2.6) as compared to the Tromsø participants (M = 1.6). This variability may stem from the mere design of the interviews where different dialect features were tested for Oslo and Tromsø in the wordlist tasks, but also from somewhat different profiles of the two L3 dialect groups.

**Table 9:** Descriptive statistics for dialect use across regions and styles (scale: 0–6).

	Wordlist	Reading	Picture description	Unscripted speech	Overall score
Oslo					
Mean	3.8	2.1	2.3	2.2	2.6
Median	3.8	1	2	2	3
SD	1.4	1.7	1.7	1.5	1.6
Min.	1.1	0	0	0	0
Max.	5.5	5	5	4	4
Tromø					
Mean	2	2	1.9	1.6	1.6
Median	1.6	1.5	1	1	1.3
SD	1.5	1.5	2.4	1.8	1.2
Min.	0	0	0	0	0
Max.	4.6	5	6	5	4.7
Joint groups					
Mean	2.9	2.05	2.1	1.9	2.1
Median	2.7	1.3	1.5	3	2.2
SD	1.45	1.6	2.0	3.3	1.4
Min.	0	0	0	0	0
Max.	4.6	5	6	5	4.7

**Figure 1:**
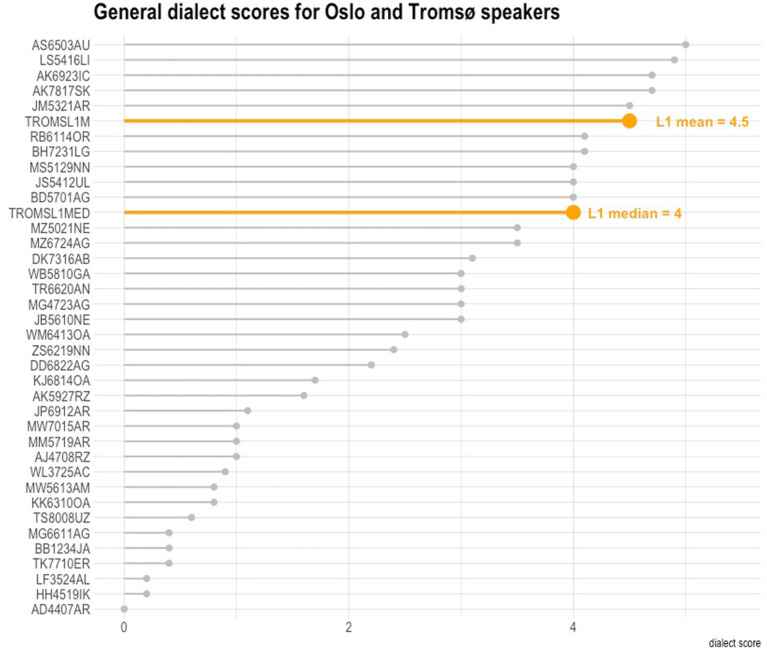
General dialect scores across all participants (L1 Norwegian scores presented as benchmark in colour).

General dialect scores were computed for all participants (see Figure 1). Each speaker of Norwegian scored between 0 and 6 points. There is only one speaker for whom we did not record any dialect features from the area (AD4407AR). This speaker was in the Oslo group, was very proficient in Norwegian (score: 27/28) but had lived for less than 2 years in Norway. The lowest dialect scorers from Tromsø were HH4519IK and LF3524AL (0.2 / 6 and 0.2 / 6). They had lived in Norway for 4 and 15 years, respectively. Speaker LF3524AL was a little less proficient in Norwegian, and in formal testing attained 14 / 28 points. Other low-scoring participants were TK7710ER and TS8008UZ, recorded in Tromsø. They were fluent speakers but had spent a lot of time in the Oslo area before moving to Tromsø, which shaped their dialect considerably. In contrast, there is only one speaker who scored 5. They were recorded in Oslo, and had lived in Norway for almost 7 years. They used the language in a professional environment, working as an interpreter.

It is interesting to see that the L1 speakers of Norwegian from Tromsø do not always produce dialect features either (their mean dialect score was 4.5). The L1 Norwegian scores are presented in Figure 1 as a benchmark for comparison. This perhaps emphasises the general formal nature of the sociolinguistic interview, but also that some traditional dialect features are also undergoing change (e.g. palatalisation, lowering of /i/ to /e/) and are less and less adopted by the younger generation. The speakers below are ordered from the most frequent to the least frequent dialect users (Figure 1). It can be noted that most speakers are in fact capable of learning and using the dialect from the area where they live, yet to varying degrees. As many as 17 speakers scored 3 or more points on average. The 10 highest-scoring dialect users featured an overall dialect score of 4 or more. The speakers who scored on average higher than the L1D1 speakers of the Tromsø dialect were JM5321AR (Tromsø) AK7817SK (Tromsø), AK6923IC (Tromsø), LS5416LI (Oslo), AS6503AU (Oslo). One may instantly notice that the Oslo speakers scored higher in this index overall than the Tromsø speakers. This is due to the fact that the Oslo forms are closer structurally (in terms of morphosyntax) to the standard written forms than in the case of the Tromsø dialect. This is also reflected in the higher scores for more formal tasks like reading wordlists, for which tasks there usually is found less vernacular than in the more spoken-oriented tasks. We discuss this in Section 8.3 below, showing that for the higher-rate dialect users this trend is often in the opposite direction.

### 6.2 Linguistic and socio-cultural predictors

**Figure 2:**
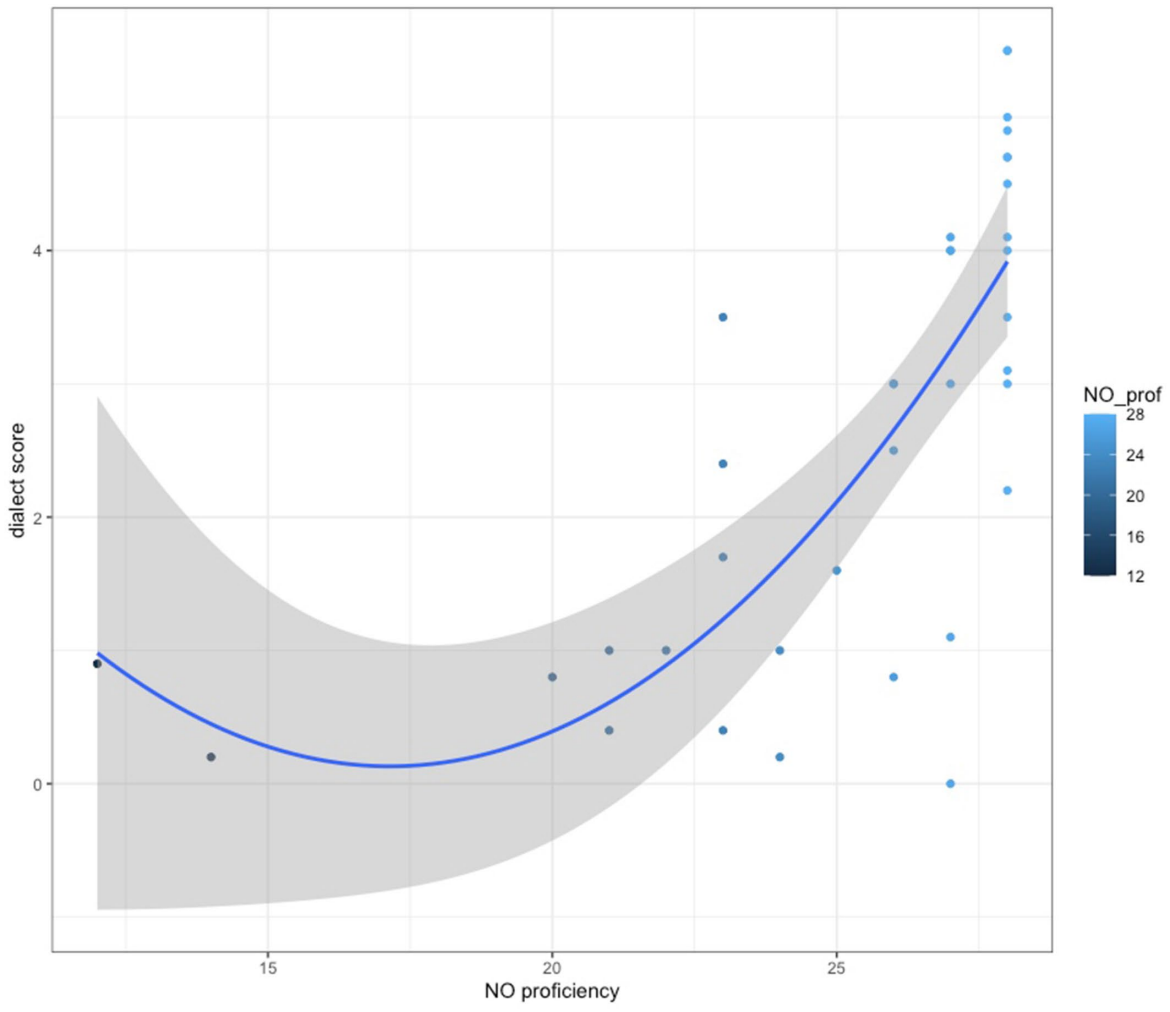
Dialect score rates against the level of Norwegian proficiency.

We tested for correlation between dialect scores and selected linguistic and socio-cultural variables in order to further explore the potential factors which play a role in dialect acquisition. Joint groups, rather than two separate groups representing each region, were entered into the analysis so that, potentially, some generalisations about dialect acquisition could be drawn. First, there was a strong positive relationship between the dialect score and the level of Norwegian proficiency at the level of significance (*r*(36) = 0.65, *p* = 0.000018). The data points are presented in Figure 2.

**Figure 3:**
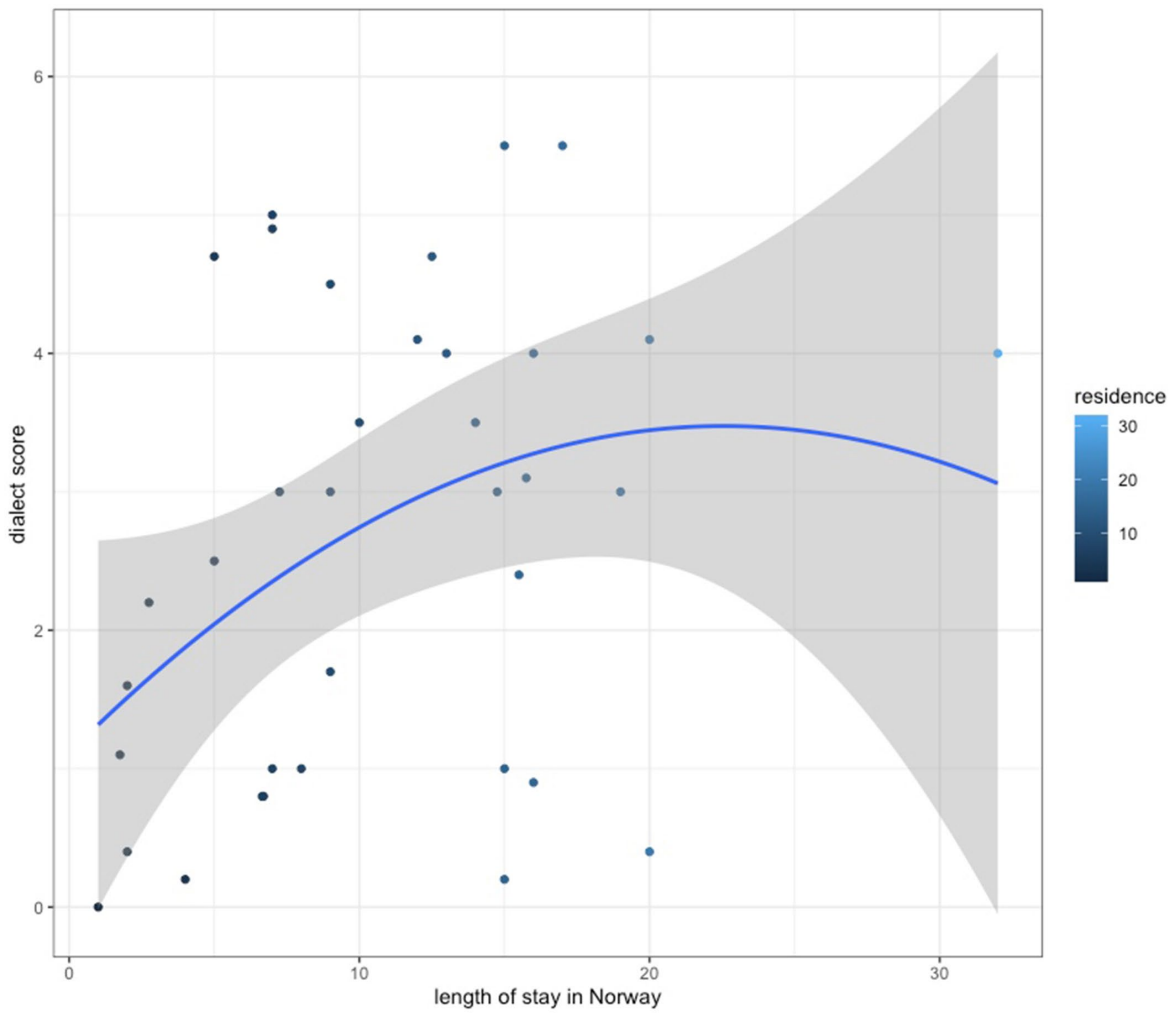
Dialect score rates against the length of stay in Norway.

As far as the measured socio-cultural factors are concerned, a parallel correlation analysis was performed. This yielded significant moderate correlation between the length of stay in Norway, expressed in years, and the dialect score (*r*(36) = 0.33, *p* = 0.049348). The results indicate that the longer the residence in Norway, the more likely the speakers of migrant backgrounds are to use dialectal features in their Norwegian speech production (see Figure 3).

**Figure 4:**
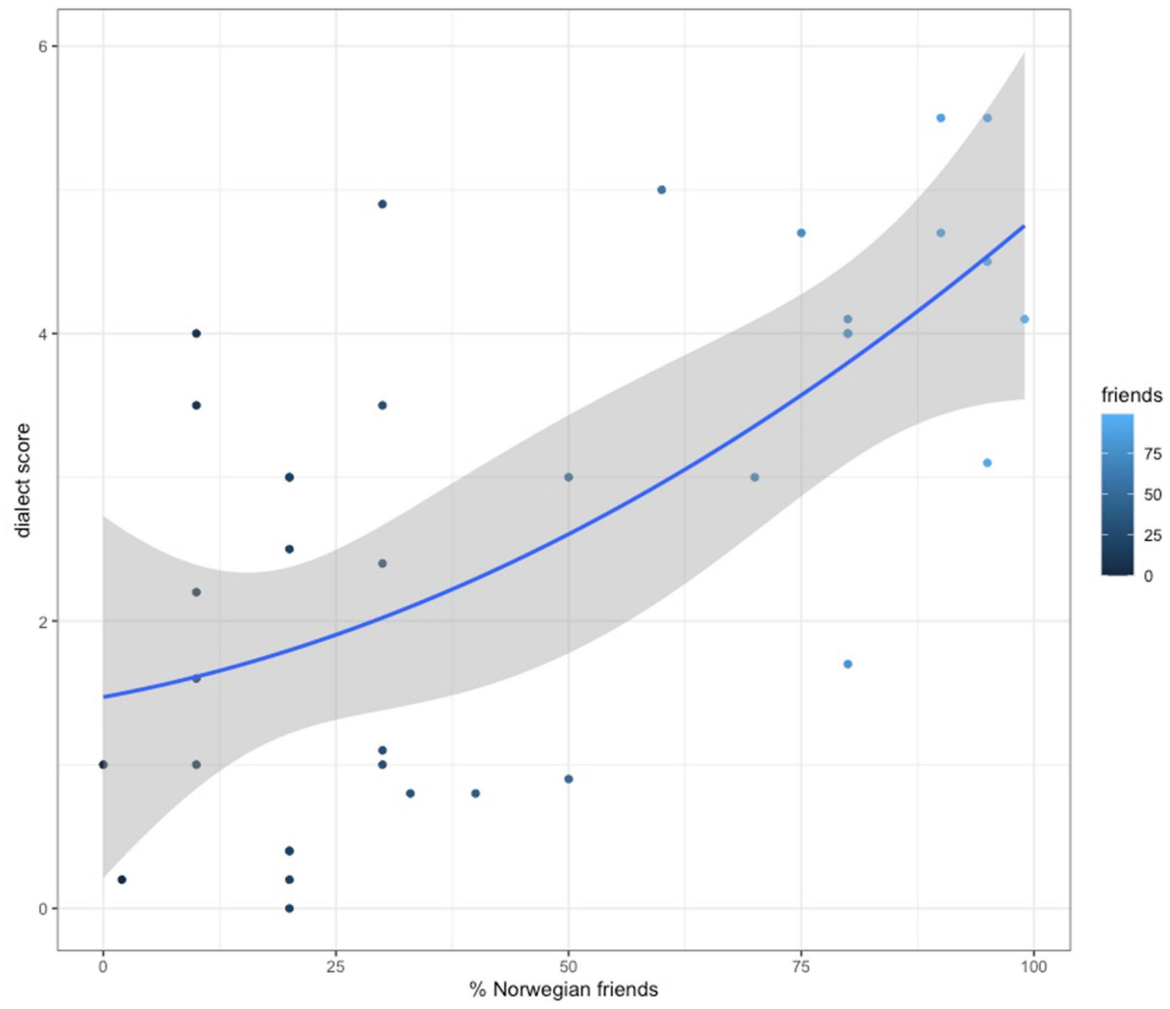
Dialect score rates against % of Norwegian friends in one’s circles.

Another striking pattern we have found was the link between general dialect scores and the number of Norwegian L1 speakers as friends in participants’ circles. There is a significant correlation of moderate strength between the dialect score and the percentage of Norwegian friends in one’s circles (*r*(36) = 0.64, *p* = 0.000026; see Figure 4).

**Figure 5:**
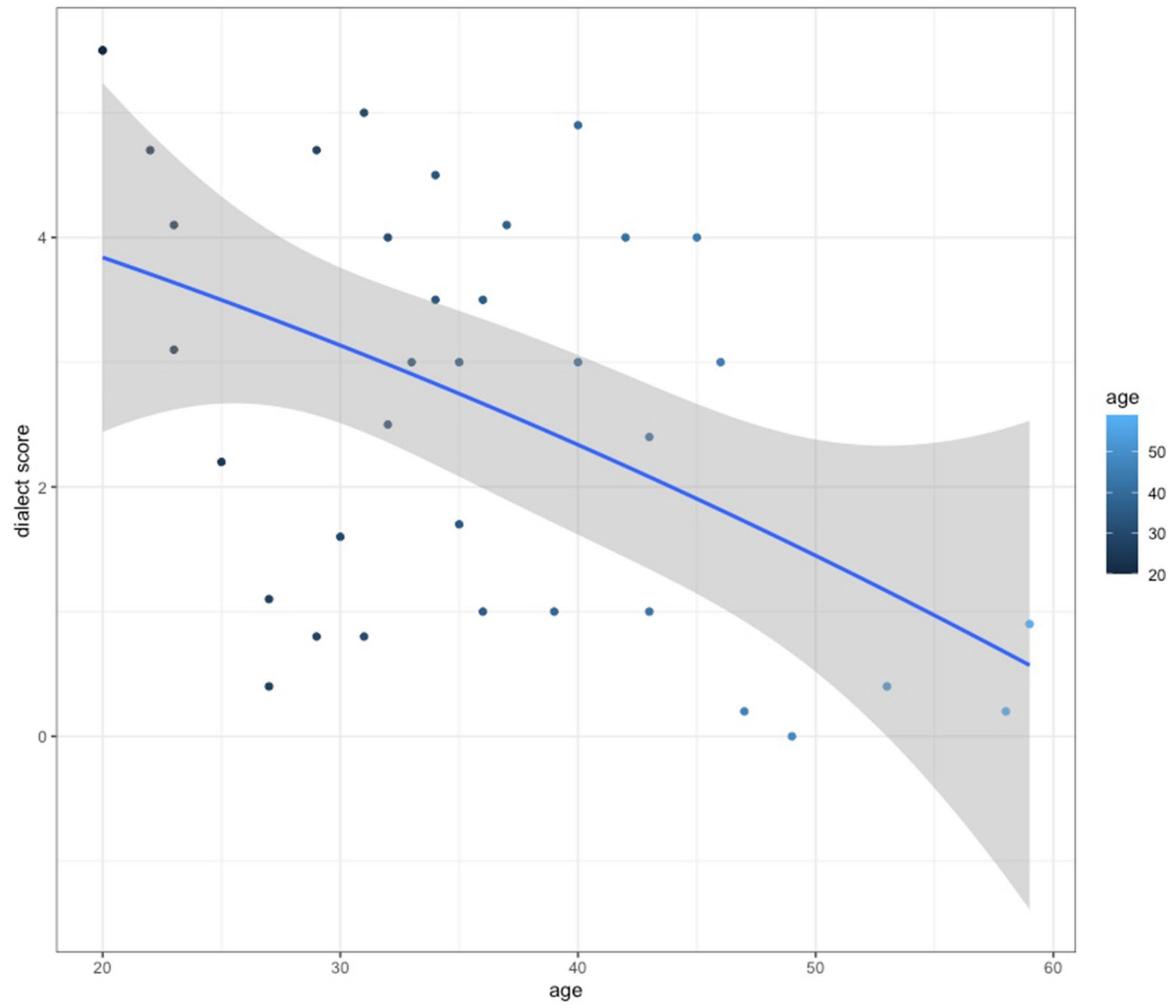
Dialect score rates against participants’ age.

Additionally, we found that general dialect scoring correlated negatively with the speakers’ age (r(36) = −0.49, *p* = 0.002417; see Figure 5). The younger the speaker, the more dialect they used. This result may be indicative of different waves of the Polish migration to Norway.

### 6.3 Style-shifting

Some speakers were found to style-shift in terms of their use of dialect features throughout the interview. For example, some speakers speak with greater fluency in the language in free speech than when they read aloud (e.g. MG4723AG, AJ4708RZ). Other speakers display the voiced retroflex flap [ɽ] (*tjukk /l/*) feature in open speech, but not when reading the wordlist (e.g. JS5412UL), and one speaker in Tromsø displayed palatalisation in words like *mann* (‘man’) when describing a picture and talking about their free time, but not when they read the wordlist (e.g. AK6923IC). Below, we present a closer examination of what exact patterns are found in style-shifting in both groups.

**Figure 6:**
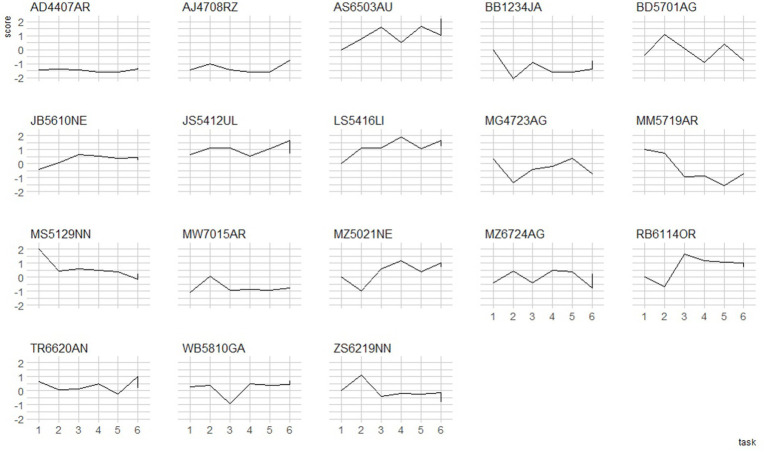
z-score plots for style shifting trajectories in the L3 Norwegian Tromsø group. 1 = wordlist, 2 = reading, 3 = picture description, 4 = unscripted (task 1), 5 = unscripted (task 2), 6 = unscripted (task 3).

**Figure 7:**
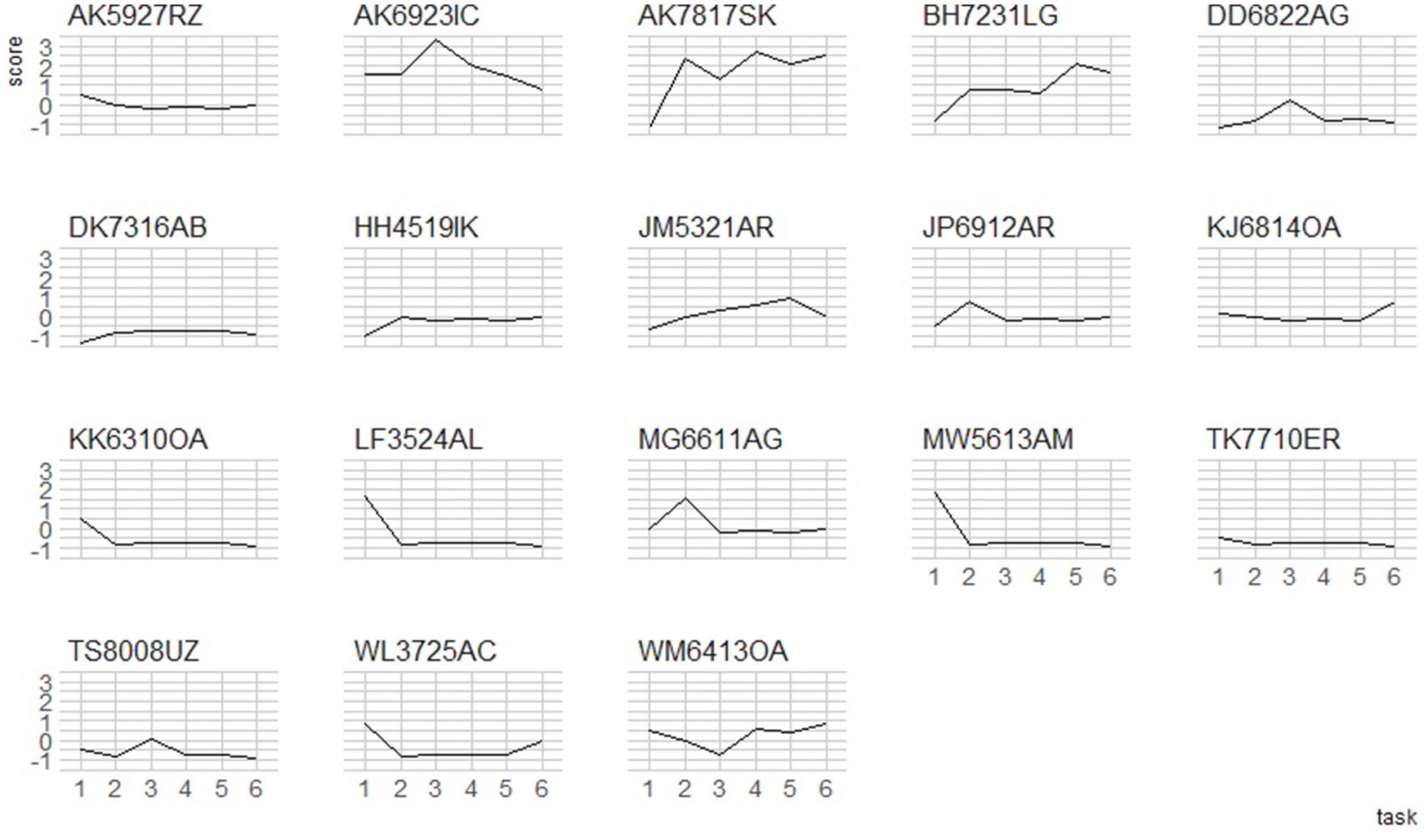
z-score plots for style shifting trajectories in the L3 Norwegian Oslo group. 1 = minimal pairs, 2 = wordlist, 3 = picture description, 4 = unscripted (task 1), 5 = unscripted (task 2).

The two figures below represent style-shifting patterns for each speaker; Figure 6 for the Oslo group, and Figure 7 for the Tromsø group. The data points are the z-transformed dialect scores for each part of the interview for each speaker, which enabled us to better plot the deviations from the average dialect scores in each participants’ performance in each task. The speech styles are presented from the most formal to the least formal speech style. The last three tasks (4, 5, 6) all represent unscripted speech. It was expected that with the first unscripted speech (semi-spontaneous) task, the interviewees would become more relaxed with every question about their hobbies, everyday life etc.

The results presented below may be interpreted in more than one way. First, style-shifting has usually been described in the context of variable use of the vernacular in different parts of a sociolinguistic interview; and this is what shows for many speakers. Namely, many high-frequency dialect users (e.g. AS6503AU, RB6114OR, MZ5021NE, TR6620AN in Oslo, and AK7817SK, BH7231LG in Tromsø) tend to display less dialect in more formal speech styles, as opposed to more casual styles. This trend was reversed for the second group (e.g. MS5129NN, ZS6219NN, MM5719AR in Oslo, and AK7817SK in Tromsø) for whom the fluctuations were recorded in the other direction, yet they still maintained the relatively high proportions for their dialect use. The third group displayed relatively constant rates for dialect use, and this most often coincided with very little dialect in their repertoire (e.g. AD4407AR, DK7316AB). However, different parts of the interview may also be treated as representative of different language modalities, i.e. read vs. spoken (Figure 7).

### 6.4 Dialect feature hierarchy

We identified all dialect features used by our informants in Oslo and Tromsø in the free speech tasks, in order to categorise them and build a dialect feature hierarchy. Let us first look at the results from the Oslo participants. Not surprisingly, there is a clear gradation in terms of how often various features are used. First, retroflexes are featured in many speakers’ L3 dialects and our data suggests that this could be the first dialect feature to be acquired by Polish learners of Norwegian when the language is learnt in the naturalistic context. Retroflexion does not appear in Polish to this degree in terms of tongue position (but there is an ongoing discussion about the phonemic - allophonic status of retroflexes in Polish, *cf.* Żygis et al., 2012), but is more frequent in Norwegian. It is not exclusively an Oslo or southeastern dialect feature, it appears in all parts of Norway, with the exception of the Western regions (Kristoffersen, 2000; Helleland and Papazian, 2005). Retroflexes appear when /r/ is followed by an alveolar or a dental consonant which gives [ɳ], [ʈ], [ɭ] etc. The second most common feature of the Oslo dialect attested in our participants’ speech was the use of the tonal opposition between low tone (*lavtone*) and high tone (*høgtone*). Here, however, we have differentiated between two different situations, i.e. one in which the feature was fully acquired and one in which speakers used it variably or infrequently. It seems that many speakers have tone 1 – tone 2 differentiation, at least in some words. This is why the category of some lavtone has been included in the analysis of the collected data to account for those L3 Norwegian speakers who are able to distinguish between the two tones, but fail to use this feature consistently. The fully acquired feature, produced in all contexts applicable, is a little less frequent but is still used by more than half of the Oslo speakers (*nota bene*, we were not testing this feature among Tromsø speakers because the tonal opposition is lost or minimal in the production of many L1D1 Tromsø speakers). The next feature in the hierarchy, as used in the interviews, is the form of the feminine nouns, where they are ascribed the indefinite article *en* (for both masculine and feminine nouns *cf.* Lødrup, 2011, Rodina and Westergaard, 2015), but the feminine ending -*a* is used as a definite article, a feature which has become common even in very conservative West Oslo dialects which traditionally did not use the feminine endings in words like *jenta* (‘girl’; Western, 1977; Mæhlum and Røyneland, 2023). In recent years, however, the use of -a endings in feminine nouns has been growing, also among speakers of the West Oslo dialect, most interestingly among young female residents (*cf.* Johannessen, 2008). This trend is reflected in the speech patterns of the Polish informants as many of our L3 Norwegian speakers have this form in their repertoire. Another Oslo dialect feature found in our participants is *tjukk /l/*, or a thick /l/, which is a retroflex flap [ɽ], used, e.g. in words like *sola*. The next Oslo-area dialect feature in the hierarchy was the replacement of long /*e*/ with a diphthong /*æi*/. Finally, there were three features which were found very infrequently in our speakers; one of these was the stress shift to the initial syllable in foreign words, a feature which is gradually disappearing among Norwegian L1 speakers and is predominantly characteristic of elder male Norwegian L1 speakers (Mæhlum and Røyneland, 2023: 61).

The hierarchy as presented in Table 10 allows us to make certain inferences about which dialectal features may be used by whom, at what frequency or in which order they are likely to be acquired. For example, if a speaker uses the voiced retroflex flap [ɽ] for /l/ (*tjukk /l/*), they most probably will have retroflexes and tonal opposition in their repertoire. The tonal opposition of Norwegian high and low tone has been acquired, though with a certain degree of inconsistency, by more than half of our Oslo speakers. This is a relatively high number for a feature which is considered especially difficult for learners of Norwegian as far as its distribution rules are concerned. It also seems that the use of feminine nouns with a definite postpositional article *-a* is a rather frequent dialect feature and, perhaps, one of the first to be acquired by learners of Norwegian living in the country. The frequency of use of these dialect features may also be indicative of how universally spread they are among L1D1 Oslo speakers. The less exposed Ln speakers are to a given feature, the less likely they are to acquire it. This is also related to the social status of each dialect feature, e.g. *tjukk /l/* (retroflex flap [ɽ]) still is a rather stereotyped dialect variant, avoided by speakers of some higher-status Oslo sociolects (Mæhlum and Røyneland, 2023: 61).

**Table 10:** Dialect feature hierarchy for the L3 Oslo speakers.

	Retroflex	Some lavtone	F	Lavtone	*Tjukk* /l/	Diph. /e/ to /æi/	tonem 1 – tonem 2 dist.	ø	-a in past tense	Initial stress
AS6503AU	✓	✓	✓	✓	✓	✓	✓	✓	✓	
LS5416LI	✓	✓	✓	✓	✓	✓	✓	✓		
JS5412UL	✓	✓	✓	✓	✓	✓		✓		
MZ5021NE	✓	✓	✓	✓	✓		✓		✓	
RB6114OR	✓	✓	✓	✓	✓		✓		✓	
MS5129NN	✓	✓	✓	✓	✓					✓
TR6620AN	✓	✓	✓	✓	✓					
WB5810GA	✓	✓	✓	✓	✓					
ZS6219NN	✓	✓	✓	✓			✓			
JB5610NE	✓	✓	✓	✓	✓					
BD5701AG	✓	✓	✓	✓						
MG4723AG	✓	✓	✓	✓						
MZ6724AG	✓	✓			✓	✓				
MW7015AR	✓	✓								
AJ4708RZ			✓			✓				
MM5719AR	✓									
BB1234JA			✓							
AD4407AR										

The Tromsø dialect features found in our participants are presented in Table 11. From the collected data it transpires that the Polish L3 speakers seem to score lower with regard to the use of dialect features than their counterparts from Oslo. As in the Oslo group discussed above, many Norwegian L3 speakers in Tromsø display retroflexion, a feature which is found in three main groups of Norwegian dialects. Among the most surprising findings from the Tromsø L3 speech samples, one can point to the high number of informants with the *tjukk* /l/ (retroflex flap [ɽ] sounds). Since *tjukk* /l/ is absent from the majority of Northern Norwegian dialects (Bull, 1996; Nesse and Sollid, 2010), one may infer that its occurrence in the speech samples of some Polish L3 speakers from Tromsø may be attributed to the fact that they moved to Tromsø from other parts of Norway where, most likely, the local dialect is marked by the presence of this sound. The Tromsø dialect is a variety of Norwegian referred to as *e/a mål* (‘e/a variety’) where infinitives have an *-e* ending, while the so-called weak feminine nouns have an *-a* ending in the indefinite form, e.g. *ei flaske*/*flaska* (‘a bottle’). The latter dialect feature is particularly relevant to the present study since as many as 14 Polish participants from Tromsø use the indefinite forms of feminine nouns with an *-a* ending. The next feature in the hierarchy is the use of Tromsø-specific personal pronouns. Particularly the first- and third-person feminine singular pronouns score very high among our participants (*æ* ‘I’ and *ho* ‘she’). Another feature to be discussed is the palatalisation of dentals, although we need to emphasise here that this dialect feature appears to be more characteristic of older speakers among L1 Norwegians (Bull, 1996; Jahr, 1996) which, in turn, may also explain why only three L3 speakers had it in their repertoire. Lastly, the varied and inconsistent realisation of the tone features in the L3 speech samples seem to attest to the fact that some participants may have learnt Norwegian in other regions where high-tone dialect features are not dominant. In Northern Norwegian dialects, the Old Norse /hv/ has become /kv/, or even /k/ in words like *kem* (‘who’), *ka* (‘what’), or *kor* (‘where’). While there might be some regional variation as regards the realisation of this onset consonant cluster (*cf.* Bull, 1996; Nesse, 2008), the low frequency of this dialect feature among the Polish participants in Tromsø may be somewhat surprising. Among the dialect features which have been traditionally ascribed to the Troms region, including the urban areas of Tromsø, (*cf.* Jahr, 1996; Johannessen et al., 2009) we can mention two, which are very infrequent in the L3 speech samples. These are *lågning* (the lowering of front vowels that occurred in the Old Norse period - *cf.* Sandøy, 1985), and palatal realisation of *ikke.* This may be explained by the findings in the Nordic Dialect Corpus (Johannessen et al., 2009) where the above features can only be traced in the speech of older L1D1 speakers of the Tromsø dialect. Finally, an infrequent feature found in unscripted speech among the Tromsø participants is the dialect form in the present forms of the strong verbs (e.g. Tromsø *kjemm(er)* for *kommer*), which has been found for only two speakers.

**Table 11:** Dialect feature hierarchy for the L3 Tromsø speakers.

	høytone	Retroflex	apocope	æ	Palatalisation	Si (gender)	dem / de	sæ	Pronouns (e.g. ho, æ)	Wh- words (kor / ka etc.)	ikkje	nokke	verb	lågning
AK7817SK	✓	✓	✓	✓	✓	✓	✓	✓	✓					✓
AK6923IC	✓	✓	✓	✓	✓				✓	✓		✓		
JM5321AR	✓	✓		✓	✓				✓				✓	✓
BH7231LG	✓	✓	✓	✓			✓			✓			✓	
DK7316AB	✓	✓	✓											
WM6413OA	✓	✓	✓											
WL3725AC			✓					✓		✓				
TK7710ER	✓	✓												
MW5613AM	✓		✓											
TS8008UZ	✓		✓											
JP6912AR		✓				✓								
DD6822AG	✓											✓		
KJ6814OA		✓									✓			
KK6310OA				✓							✓			
MG6611AG						✓	✓							
AK5927RZ	✓													
HH4519IK	✓													
LF3524AL														

In order to be able to compare the acquisition patterns of various dialect features, the following hierarchy of the dialect forms has been built for the L1 speakers recorded in Tromsø (Table 12). Once again, like for the L3 speakers above, the most frequently featured features come first (the left-hand columns), and are presented in the descending order. The rows, in turn, are sorted in the descending order in terms of the number of dialect features found in each speaker, i.e. the most Tromsø dialect features were recorded for GM7215OR, and the least for JRM6431UG. The dialect feature which appeared in speech of all L1 participants were retroflex sounds. The next in frequency were *høytone* (high tone), *æ* as the 1st pers. singular form, and palatalisation. It came as no surprise that the L1D1 speakers used more dialect features than the Ln speakers inhabiting the same region in Norway. However, we found no instances of the following three features in fact recorded for the L3 speakers: *nokke*, *dem / de* and the feminine form of the possessive pronoun *si*.

**Table 12:** Dialect feature hierarchy for the L1 Tromsø speakers.

	Retroflex	høytone	æ	Palatalisation	Wh- (kor / ka etc.)	Plural forms	lågning	kjeklet	Sæ / maæ	ikkje	verb	Pronouns (e.g. ho)	-a in past tense	oppdaga
GM7215OR	✓	✓	✓	✓	✓		✓	✓	✓		✓		✓	✓
HA5809AR	✓	✓	✓	✓	✓	✓	✓	✓			✓		✓	
KF2804NN	✓	✓	✓		✓	✓	✓	✓		✓	✓		✓	
GE5012ER	✓	✓	✓	✓	✓		✓	✓	✓	✓				✓
ER6615MA	✓	✓	✓	✓		✓	✓	✓	✓	✓				
BR6119AN	✓	✓		✓	✓									
EF5520UR	✓		✓	✓	✓	✓		✓	✓			✓		
JRM6609ET	✓	✓	✓		✓			✓	✓		✓	✓		
GFL5224AI	✓	✓	✓	✓		✓	✓	✓						
KK2725EL	✓	✓	✓	✓		✓		✓				✓		
PR4819IR	✓	✓	✓	✓		✓		✓		✓				
KH5216OH	✓	✓	✓	✓			✓	✓						
JRM6431UG	✓	✓	✓					✓						

### 6.5 Individual variability

The patterns for dialect shifting, despite the generalisations drawn above, vary between different speakers. The degree of inter-speaker variation, however, is high among both L3 and L1 Norwegian speakers. Looking into several individual patterns for L3 dialect acquisition may help provide a more nuanced understanding of this process, and hence a few examples will be presented below.

A good example of a fully immersed dialect learner is speaker AK6923IC, recorded in Tromsø. At the time of the interview they had spent around 5 years in the area. They were a young person, close to finishing their university degree. They had learnt Norwegian in a naturalistic setting, speaking to their co-workers and friends. They were a fully fluent speaker, with a vast majority (90% reported in the questionnaire) of their friends being Norwegian. Their partner was Norwegian, too. Most of the time, they also used Norwegian for everyday communication. Their performance is a good example of how a high dialect user orients themselves towards the use of the vernacular. It seems that their pragmatic processing of the dialect features is similar to what would be expected from an L1 speaker from the region. In the unscripted speech tasks, we were able to detect a number of Tromsø dialect features in their performance, including the regionally used pronouns (e.g. *ho* for *hun*) or palatalisation of the word-final */l/, /n/, /k*/ (e.g. in the word *hund*; Supplementary Audio 1). They also displayed considerable style-shifting, e.g. there is none or much less palatalisation in the wordlist reading (e.g. in *kann, man*; Supplementary Audio 2). They were a fully fluent Norwegian speaker (Supplementary Audio 3).

Let us now look at a Norwegian spoken by AJ4708RZ. This speaker was recorded in Oslo. They had spent a lot of time in the area, working as an engineer. They were a low dialect scoring participant. One interesting thing to be noticed about their language acquisition pattern is that they seem to be struggling with the reading tasks, reading rather slowly as if they were not a proficient language user (Supplementary Audio 4), while clearly speeding up and feeling more comfortable when speaking about their free time (Supplementary Audio 5). Their speech becomes more fluent, as if indicating how their language learning process could have been completed, namely, perhaps in a naturalistic environment at the expense of classroom instruction and the experience of reading in Norwegian. Even though they are rather fluent in the open speech tasks, their accent is retained as very L1-driven, or heavily Polish sounding. In the open speech tasks, we were able to detect only two Oslo dialect features, i.e. the diphthongisation of /e/ to /æi/, and the Oslo-like forms of the feminine nouns. In terms of their socio-economic profile, they have settled in the Oslo area and are very stable economically. They seem to be a speaker assimilated within society considering their family and work status, however, they reported not having any L1 Norwegian friends in their circles which may have an impact on the acquisition of their dialect. What is also interesting is that their accent skills in English seem impressionistically comparable to their accent skills in Norwegian (Supplementary Audio 6).

In Tromsø, we recorded a subset of speakers who had spent considerable time in South-East Norway before moving to the Tromsø area. This was clearly represented in their dialect scores. Since they were permanent residents of Tromsø for a longer time, we still wanted to measure to what extent they may acquire the Tromsø dialect, or be prone to employ the strategies for dialect accommodation. In fact, we did not find many Tromsø dialect features. *Kjekla* (apocope), the use of *ikkje*, and the employment of high tone from time to time, were featured in the speech of these participants. This signals that dialect accommodation, or second dialect acquisition in an Ln, may not be a likely linguistic behaviour among similar communities living in Norway, or that it may be less likely for a person to accommodate to a dialect lower on the prestige scale (here, Tromsø) from a dialect of a higher perceived relative prestige (here, Oslo).

## 7 Discussion

In the first research question we wanted to investigate which varieties of the Norwegian language are spoken within the circles of Polish-born Norwegian inhabitants, comprising the largest migrant group in the country. It turned out that most of the recorded speakers did indeed use at least some dialect features from the region where they lived. Their dialect scores differed but this was predictable given that the dialect is variably used in sociolinguistic interviews because of their inherent design eliciting the vernacular to different degrees in its different parts. This result was also mirrored in the performance of the control group, i.e. L1 Tromsø speakers, for whom large variation was also recorded.

Another finding was that the level of dialect use was dependent on the language mode. Namely, for many speakers, especially the more frequent dialect users, the unscripted speech tasks where the participants were asked about their daily lives were more conducive to the use of dialect. Some speakers in Tromsø, for instance, palatalised all or some words when spontaneously answering the open questions (e.g. in the words *hund*, *mann*, *kann*) but they did not have this feature in reading, resorting to the standard nasal sound /n/. When listing down the dialect feature repertoire for each speaker (see Tables 10, 11), we focused only on the material used in the unscripted speech tasks. Building a hierarchy of dialect features, we believe, gives insight into which features are more frequently used and perhaps also acquired first in the process of dialect acquisition. This addresses the second research question: participants do seem to develop a sensitivity towards the dialect and use it differently in different speech registers.

The third research question focused on variability in dialect use along social and linguistic variables. We found a set of positively correlating factors which were the level of proficiency in Norwegian, the length of stay in the region (as opposed to length of stay in Norway overall), and the percentage of Norwegian L1 speakers present in one’s circles. We analysed all the speakers, from both regions, collectively in these analyses, in order to present the potential predicting factors for dialect acquisition which would be universal, irrespective of the target dialect.

The patterns for style-shifting differ between the Oslo and the Tromsø speakers. This may be due to the design of the interview itself, but undoubtedly the status of the Oslo dialect plays a role, too, in the way that many forms used are closer to the written standard than in the case of the Tromsø dialects. One more potentially relevant factor is the clearly different sociodemographic profiles of the speakers coming from the two regions. The Tromsø participants had lived on average for a shorter time in the country and, notably, almost half of them had reported living somewhere else in Norway. This probably explains, e.g. the retroflex qualities which sounded as if acquired in a different region, as reported in a few participants. Although all the Tromsø speakers had met the recruitment criteria (having lived for longer in Norway and in the region), we did record speakers with more South-Eastern sounding accents. We did not exclude them as outliers, especially because they met the inclusion criteria for this study. Instead, we measured whether there were any Tromsø dialect features that may have developed in their repertoire. It was interesting to investigate which features would be acquired first for someone who had lived in the South-East of Norway. In a conversation outside of the recorded interview with TK7710ER, when asked if they thought they had any Tromsø features in their speech, they reported the lowering of the vowel /*i*/ to /*e*/ in their speech sometimes, and gave example of the word *fisk* (‘fish’). They said this accent feature was the first feature that they noticed upon moving to the area. Overall, however, it must be concluded that these speakers have not yet accommodated their speech to the dialect spoken in the North. There are not many studies on second-dialect acquisition in a foreign language (L2D2), but Gnevsheva et al. (2022) suggested that dialect accommodation may be quicker in the second language than in the first.

Given the different status of the two dialects, one alternative interpretation of the style-shifting results could be given. Because the Oslo dialect is understood as the closest variety to the standard Norwegian speech, there would be little possible variability between the dialect and the standard forms. The fluctuations in the dialect score results then could mirror image different speech modalities, i.e. read vs. spoken. One more aspect which is different in describing style-shifting patterns between L1 and L3 speakers is also that L3 speakers have one more modality into which they may shift into, i.e. their L1 categories. For example, for those speakers who did not display retroflexion in both regions, the alternative form was not the standard, but rather their Polish-influenced pronunciations.

This data may, therefore, also point to some regularities in the acquisition of Norwegian in general. For example, many of our participants display retroflexion (which is of a different quality than in their first language). This could mean that the feature is easily perceptible by learners of Norwegian, or that it is mentioned during classroom instruction and incorporated relatively easily. Some prosodic features which are not found in speakers’ L1 were also present, such as the use of *lavtone* (low tone) and *høgtone* (high tone), which we did not expect to find to such a degree.

In the light of this discussion, we interpret that dialect can be acquired by L3 speakers of Norwegian. Our data shows that L3 dialect acquisition is attainable, along with sensitivity towards the vernacular and a subconscious understanding that most speakers use the dialect to various degrees. This also signals that the process belongs to the pragmatic processing of the language. The process, however, is complex, and not every L3 speaker, no matter how fluent in a foreign language (here, Norwegian), will be able to develop such a sensitivity. There were speakers (e.g. AK6923IC, AK7817SK) who use the dialect and code-switch between different tasks, as a strategy of orienting themselves towards the vernacular. What is interesting is that not all Norwegian speakers researched in Tromsø did in fact style-shift. Some used very few dialect features in their speech. This brings us once more to the notion of D1 and D2 acquisition in a first language, which is still a largely underresearched topic. Namely, some L1 speakers, just as Ln speakers, will not develop a lot of dialect, and will not be willing or capable of developing a second variety, in order to code switch or style shift in their L1. There are clearly certain cognitive and social mechanisms responsible for this (Oschwald et al., 2018), but L1 standard language ideologies and the expectations that dialects are stigmatised *per se* must also play a role (see also Auer and Røyneland, 2020).

## 8 Limitations

Perhaps one of the caveats behind this work is that the status of the two dialect regions compared is not exactly the same. The Tromsø dialect is a Northern dialect perceived differently in terms of prestige and of different typological structure than the Oslo dialect. Our aim initially was precisely to make use of these dissimilarities, in order to assess the potential differences in how the dialects may be learnt by Ln speakers. It could be argued, however, that the Oslo dialect is too often understood as the standard (or more standard than the Tromsø dialect), and hence the differences observed were unavoidable. The Oslo forms are structurally much closer to the Standardtalemål forms, and there are simply more forms in Tromsø which are divergent from the standard written forms. Hence the different results in dialect scoring for the two regions. Another is that we have found some complexity in participants’ profiles in terms of places of residence. While most Oslo participants had lived mostly in Oslo and in the larger Oslo area, about half of the Tromsø participants had lived in other dialect regions at least for some time. They still met the inclusion criteria, having settled and lived in the Tromsø area, but the few examples reported show that speakers with these characteristics did not yet accommodate their accent to the new place. On the other hand, migration within the country has been considerable in Norway for a longer time now which undoubtedly has consequences for the development of Norwegian dialects and how they are perceived (Røyneland and Jensen, 2020; Sætermo and Sollid, 2021).

## 9 Conclusion

There is no one single answer to the question of how exactly the process of dialect acquisition develops in an L2 or L3. Our data coming from migrant communities speaking Norwegian in Norway points to a lot of inter- and intra-speaker variation. There are regularities, however, and therefore, this process is not entirely idiosyncratic. Especially, there are some linguistic and extra-linguistic predictors for high and low dialect use, such as the level of Norwegian proficiency, and length of residence in Norway. We demonstrate that many participants engage in style-shifting as a pragmatic strategy, using the dialect to different degrees depending on how informal the given speech act is. The implications of these findings may point to the fact that one is ready to fully assimilate with language communities in a foreign country only after understanding how sociolinguistic variation works in a foreign language; yet trying to assimilate with such communities perhaps enforces and accelerates the process of acquiring an understanding of sociolinguistic variation, too.

## Data availability statement

The datasets presented in this study can be found in online repositories. The names of the repository/repositories and accession number(s) can be found at: https://github.com/kmalarski-amu/Malarski_et_al_2013.

## Ethics statement

The studies involving humans were approved by Adam Mickiewicz University Ethics Committee. The studies were conducted in accordance with the local legislation and institutional requirements. The participants provided their written informed consent to participate in this study. Written informed consent was obtained from the individual(s) for the publication of any potentially identifiable images or data included in this article.

## Author contributions

KM: Conceptualization, Data curation, Formal analysis, Investigation, Methodology, Project administration, Resources, Visualization, Writing – original draft, Writing – review & editing. WA: Formal analysis, Investigation, Validation, Writing – original draft, Writing – review & editing. MW: Conceptualization, Funding acquisition, Resources, Supervision, Validation, Writing – review & editing. CC: Conceptualization, Data curation, Formal analysis, Investigation, Methodology, Resources, Software, Writing – original draft, Writing – review & editing. IJ: Conceptualization, Investigation, Methodology, Writing – review & editing.

## Footnotes

^1^It could also be argued that both Bokmål and Standardtalemål are terms better stuited for describing the morphosyntactic structures, while Urban Eastern Norwegian the term inclusive of lexis and phonology, too. Undoubtedly, Urban Eastern Norwegian is what people across the country would be most acquainted with.
