# Supplementary material for: Orientation towards the vernacular and style-shifting as language behaviours in speech of first-generation Polish migrant communities speaking Norwegian in Norway
Source: Front Psychol. 2024 Aug 14;15:1330494. doi: 10.3389/fpsyg.2024.1330494 (PMC11351563; doi:10.3389/fpsyg.2024.1330494)
Supplement: Supplementary file 1 [file Data_Sheet_1.docx]

Supplementary Material

Orientation towards the vernacular and style-shifting as language behaviours in speech of first-generation Polish migrant communities speaking Norwegian in Norway

**Kamil Malarski*, Chloe Castle, Helene Ruud Jensberg, Witosław Awedyk, Magdalena Wrembel, Isabel Nadine Jensen**

*** Correspondence:** Corresponding Author: kamil.malarski@amu.edu.pl

# Sociodemographic survey questions

1. Jaki jest Twój język ojczysty?
2. Oceń stopień Twojej znajomości poniższych języków. (b. sɫaby, sɫaby, podst., średni, dobry, b. dobry, biegɫy)
   1. Polski
   2. Angielski
   3. Norweski
3. W jakim wieku zaczęłaś / zacząłeś posługiwać się poniższymi językami? (0-2 lata, 3-6 lat, 7-10 lat, 11 - 14 lat, 15 - 21 lat, 22 - 35 lat, 36+)
   1. Polski
   2. Angielski
   3. Norweski
4. Czy znasz jeszcze jakieś języki w mowie lub piśmie? Wypisz i sprecyzuj. Oceń poziom zaawansowania.
5. Od jak dawna mieszkasz w Norwegii? (w latach i miesiącach)
6. W jakich regionach lub miastach mieszkałaś / mieszkałeś wcześniej w Norwegii? Przez jak długo?
7. Jak uczyłaś / uczyłeś się języka norweskiego?
   1. formalnie (np. na zajęciach, w szkole językowej, z nauczycielem itp.)
   2. naturalistycznie (w Norwegii: w pracy, wśród znajomych, oglądając lub słuchając mediów itp.)
   3. w formie mieszanej (formalnie + naturalistycznie)
8. W jakim dialekcie języka norweskiego mówisz? Czy zmieniasz dialekt w zależności od sytuacji?
9. Jakim standardem (lub dialektem) pisanym języka norweskiego się posługujesz?
10. Poziom znajomości norweskiego Twojego partnera / partnerki:
    1. bardzo sɫaby
    2. sɫaby
    3. podst.
    4. Średni-zaawansowany
    5. zaawansowany
    6. Biegɫy
    7. jest Norwegiem / Norweżką
    8. N/A
    9. Other
11. Jakim dialektem j. norweskiego posługuje się Twój partner / Twoja partnerka? (Napisz "N/A" jeśli nie dotyczy)
12. W jakich proporcjach używasz poniższych języków na co dzień? (pasywnie: słuchając, czytając) (1 – nigdy, 2 – bardzo rzadko, 3 – rzadko, 4 – czasami, 5 – często, 6 – bardzo często, 7 – zawsze)
    1. Polski
    2. Angielski
    3. Norweski
13. W jakich proporcjach używasz poniższych języków na co dzień? (aktywnie: mówiąc, pisząc)  (1 – nigdy, 2 – bardzo rzadko, 3 – rzadko, 4 – czasami, 5 – często, 6 – bardzo często, 7 – zawsze)
    1. Polski
    2. Angielski
    3. Norweski
14. Jak bardzo podoba Ci się język norweski? (1 – zupełnie mi się nie podoba; 7 – bardzo mi się podoba)
15. Jak bardzo podoba Ci się dialekt z Tromsø? (1 – zupełnie mi się nie podoba; 7 – bardzo mi się podoba)
16. Co sądzisz o dialekcie norweskiego z Tromsø?
17. Jak często posługujesz się dialektem z Tromsø, gdy posługujesz się językiem norweskim? (1 – nigdy, 2 – bardzo rzadko, 3 – rzadko, 4 – czasem, 5 – często, 6 – bardzo często, 7 – zawsze)
18. W jakim stopniu rozumiesz dialekt z Tromsø?
    1. wcale
    2. bardzo słabo
    3. słabo
    4. trochę
    5. dobrze
    6. bardzo dobrze
    7. wyszystko
19. W jakim stopniu rozumiesz dialekt z Oslo?
    1. wcale
    2. bardzo słabo
    3. dłabo
    4. trochę
    5. dobrze
    6. bardzo dobrze
    7. wyszystko
20. W jakim zawodzie pracujesz?
21. Zawody/Profesje/Prace Twojego pierwszego opiekuna, np. ojca (teraz i w przeszłości):
22. Zawody/Profesje/Prace Twojego drugiego opiekuna, np. matki (teraz i w przeszłości):
23. Jaki procent Twoich znajomych i przyjaciół w Norwegii to Norwegowie?
24. Jak często wracasz / jeździsz do Polski? (nigdy, bardzo rzadko, rzadko, czasem, często, bardzo często, zawsze)
